# Identification of Novel Predictor Classifiers for Inflammatory Bowel Disease by Gene Expression Profiling

**DOI:** 10.1371/journal.pone.0076235

**Published:** 2013-10-14

**Authors:** Trinidad Montero-Meléndez, Xavier Llor, Esther García-Planella, Mauro Perretti, Antonio Suárez

**Affiliations:** 1 The William Harvey Research Institute, Barts and The London School of Medicine, Queen Mary University of London, London, United Kingdom; 2 Digestive Diseases and Nutrition Section, Department of Medicine and Cancer Center, University of Illinois at Chicago, Chicago, United States of America; 3 Hospital Santa Creu i Sant Pau, Universitat Autónoma de Barcelona, Barcelona, Spain; 4 Centro de Investigación Biomédica, Universidad de Granada, Armilla, Spain; University of Torino, Italy

## Abstract

**Background:**

Improvement of patient quality of life is the ultimate goal of biomedical research, particularly when dealing with complex, chronic and debilitating conditions such as inflammatory bowel disease (IBD). This is largely dependent on receiving an accurate and rapid diagnose, an effective treatment and in the prediction and prevention of side effects and complications. The low sensitivity and specificity of current markers burden their general use in the clinical practice. New biomarkers with accurate predictive ability are needed to achieve a personalized approach that take the inter-individual differences into consideration.

**Methods:**

We performed a high throughput approach using microarray gene expression profiling of colon pinch biopsies from IBD patients to identify predictive transcriptional signatures associated with intestinal inflammation, differential diagnosis (Crohn’s disease or ulcerative colitis), response to glucocorticoids (resistance and dependence) or prognosis (need for surgery). Class prediction was performed with self-validating Prophet software package.

**Results:**

Transcriptional profiling divided patients in two subgroups that associated with degree of inflammation. Class predictors were identified with predictive accuracy ranging from 67 to 100%. The expression accuracy was confirmed by real time-PCR quantification. Functional analysis of the predictor genes showed that they play a role in immune responses to bacteria (PTN, OLFM4 and LILRA2), autophagy and endocytocis processes (ATG16L1, DNAJC6, VPS26B, RABGEF1, ITSN1 and TMEM127) and glucocorticoid receptor degradation (STS and MMD2).

**Conclusions:**

We conclude that using analytical algorithms for class prediction discovery can be useful to uncover gene expression profiles and identify classifier genes with potential stratification utility of IBD patients, a major step towards personalized therapy.

## Introduction

Crohn’s disease (CD) and ulcerative colitis (UC), the two main clinical forms of IBD, are chronic relapsing inflammatory disorders of the gastrointestinal tract resulting from an inappropriate and continuous inflammatory response to commensal microbes in genetically susceptible hosts, although the exact etiology remains unknown [1]. CD and UC are distinct but heterogeneous entities, frequently showing overlapping symptoms/features with systemic and extra-intestinal complications that render the diagnosis difficult and inaccurate. Furthermore, the degree of heterogeneity within both forms of IBD is also high, and patients with the same diagnosis, i.e. CD or UC, may have markedly different outcomes. Most importantly, the response to therapy cannot be predicted at diagnosis and therapeutic approaches are individualized according to the symptomatic response and tolerance to medical intervention. Previous attempts to identify prognostic markers have focused on clinical factors that lack specificity and, hence are not clinically useful [2]. In the absence of unique biomarkers of disease activity, severity is established based on clinical parameters, systemic manifestations, and the global impact of the disease on the patient’s quality of life [3]. Therapeutic options are determined by assessing disease location, severity, and extra-intestinal complications. Therapy approaches are based on either a “step-up” management strategy by increasing immunosuppression or a “top-down” alternative with the early use of anti-TNF-alpha treatment. In both cases, economic and safety concerns exist regarding the indiscriminate use of these strategies due to the elevated cost of anti-TNF-alpha treatment, potential unnecessary immunosuppression, life-threatening side-effects and long-term sequelae of drug treatment [4].

As a consequence of the heterogeneity of IBD patients, a comprehensive evidence-based search of novel biomarkers for an appropriate patient stratification that account for the inter-individual differences in severity, drug efficacy, side effects or prognosis would help guide clinicians in the management of patients with IBD and represent a major step towards personalized medicine. Although immunological markers like anti-*Saccharomyces cerevisiae* antibodies or genetic variants of NOD2 are statistically associated with prognosis of surgical intervention in IBD [5], these markers often lack sensitivity or are not frequent enough to be clinically useful. The search of new biomarkers of IBD course can benefit from molecular approaches like gene expression profiling [6], which has successfully been used and translated into clinical practice. Van de Vijver *et al* discovered a 70-genes prognosis classifier of the clinical outcome of breast cancer patients [7]. In ovarian cancer, Bonome *et al* defined a predictor gene set of survival [8]. With the same strategy, Mckinney *et al* identified a gene expression signature with prognosis predictor value for antineutrophil cytoplasmic antibodies-associated vasculitis [9].

These studies reflect the intense effort on the development of new tools and algorithms that go far beyond the classical selection of a number of up- or down- regulated genes to reliably translate mathematical and statistical analyses into biological meaning. Accordingly, we tested a novel analytical approach [10] to study the gene expression signature of intestinal tissues from IBD patients aiming at identifying new candidate prognostic genes associated to clinically relevant parameters. We hypothesized that correlation with clinical prognostic parameters may group samples with similar gene expression profiles and help identify potential biomarkers useful for IBD stratification with higher reliability.

## Methods

### Ethics statement

Individuals were recruited either at Hospital Santa Creu i Sant Pau (Barcelona, Spain) or at the University of Illinois Hospital (Chicago, IL) after a written informed consent was obtained. The Institutional Review Board of the University of Illinois at Chicago and the Comité Ético de Investigación Clínica del Hospital Santa Creu i Sant Pau approved the protocols.

### Patients

For this study we enrolled 15 patients with an established diagnosis of ulcerative colitis, 13 patients with Crohn’s disease and 7 healthy individuals with a normal colon referred for colorectal cancer screening. Disease activity was assessed based on parameters such as: abdominal pain, bowel consistency and frequency, blood in stool, nausea/vomiting, constitutional symptoms, extracolonic manifestations, presence of abdominal mass, blood inflammatory markers, and colonic biopsy results. Other data collected includes: age at diagnosis, disease extension, disease-related surgery, tobacco consumption, co-morbidities, medications, and need of or response to immunomodulators (Azathiopurine or 6-Mercaptopurine), Infliximab, and glucocorticoids. A summary of these data is shown in Table S1. Patients were classified as glucocorticoid-dependent when they responded to glucocorticoids but experienced loss of clinical response when glucocorticoids were tapered to less than 30 mg/day or less, or relapse within the first 3 months after glucocorticoids are stopped. Patients were classified as glucocorticoid-resistant when there was a lack of meaningful clinical response to glucocorticoids up to doses of prednisone 1 mg/kg/day (or equivalent) within 30 days for oral therapy or 7 days for IV therapy. Patients were purposefully selected for this study to represent different clinical phenotypes.

### Isolation of RNA and microarray hybridization

Four endoscopic pinch biopsies of macroscopically un-inflamed mucosa were taken from ascending colon. One biopsy was fixed in 4% buffered formaldehyde. Total RNA was extracted from three pooled biopsies using the RNeasy Micro kit (Qiagen, Crawley, UK)**.** All sample labeling, hybridization, staining and scanning procedures were carried out using Affymetrix standard protocols and equipment (Affymetrix, Santa Clara, CA). Briefly, double-stranded cDNA was synthesized from 2μg of total RNA using the One-Cycle cDNA Synthesis kit. *In vitro* transcription was performed using the IVT Labelling kit and the cRNA obtained was checked for quality and purified with the GeneChip Sample Cleanup Module. 15μg of cRNA were fragmented and hybridized on Human Genome U133 plus 2.0 arrays for 16 hours at 45°C. Arrays were then washed and stained with streptavidin-phycoerythrin and images scanned and analyzed with Affymetrix GeneChip Command Console (AGCC 1.1). Spike controls (BioB, BioC, BioD and Cre) were used as quality controls. Following MIAME recommendations, microarray data were deposited in Gene Expression Omnibus database (accession number GSE36807) where protocols, normalization methods and original raw data are publicly available.

### Data normalization, filtering and clustering

Bioconductor software version 2.15 was used for data normalization (affyPLM package, RMA method) and quality controls (% present genes, average background, scale factor GAPDH 3’/5’ ratio and spike-in controls; simpleAffy package). Data analysis and filtering were made with Spotfire DecisionSite v9.0. Normalized intensity values were used to obtain the fold change values, calculating the log_2_ of the ratio [average of the cases replicates/average of the control replicates]. Genes with a fold change >1 or <−1 and p<0.05 (t-test) were considered differentially expressed. Hierarchical clustering (Unweighted Pair Group Method with Arithmetic Mean and Euclidean distance similarity measure) and principal component analysis were also performed with Spotfire DecisionSite v9.0.

### Functional analysis and predictor discovery

Functional analyses of differentially expressed genes were made using Panther Classification System v6.1 [11]. We used the tool Prophet [10] to find the optimal subset of genes that best distinguish between the experimental groups, the class predictor genes. Predictors were built with the 54,675 probes contained in the microarray. Briefly, the software randomly divides the data in partitions, chooses one partition as training test set and builds several predictors whose efficiency is checked with the training set at a time. The random splitting of samples significantly increases predictive accuracy and self-validates the results. This process is repeated as many times as the number of partitions. Then the error is calculated for each of these predictors and, finally, the smallest set of genes that results in the smallest error is reported. The *F* ratio test was selected for the ranking of genes. The five algorithms implemented in Prophet were tested in all comparisons: support vector machine, *k*-nearest neighbor, diagonal linear discriminant analysis (DLDA), self-organizing maps and nearest shrunken centroids. DLDA generated predictor sets with the lower classification error rates. A leave-one-out cross-validation strategy was performed to find the best predictor with the optimal number of genes.

### Quantitative real time-PCR validation

cDNA was synthesized using 1μmg of total RNA with the SuperScript III Reverse Transcriptase (Invitrogen, Paisley, UK). Real time-PCR was performed with 200 ng of cDNA per well and Power SYBR Green PCR Master Mix (Applied Biosystems, Warrington, UK), using the ABI Prism 7900HT Sequence Detection System. The full list of Quantitect primers (QIAGEN, Crawley, UK) used is reported in Table S2. Dissociation step was included to confirm the absence of un-specific products. Data (n = 3) is reported as ΔCt using GAPDH as endogenous control.

### Statistical analysis

Microarray data was analyzed using t-test. Clinical data was analyzed by Fisher’s exact test or Chi-square test for trend when appropriate. Real time-PCR was analyzed by Pearson correlation and linear regression tests. Predictors were analyzed by two-way ANOVA. In all cases *p*<0.05 was considered statistically significant.

## Results

### Microarray data analysis groups high and low inflammation subclasses of CD and UC patients

**Figure 1:**
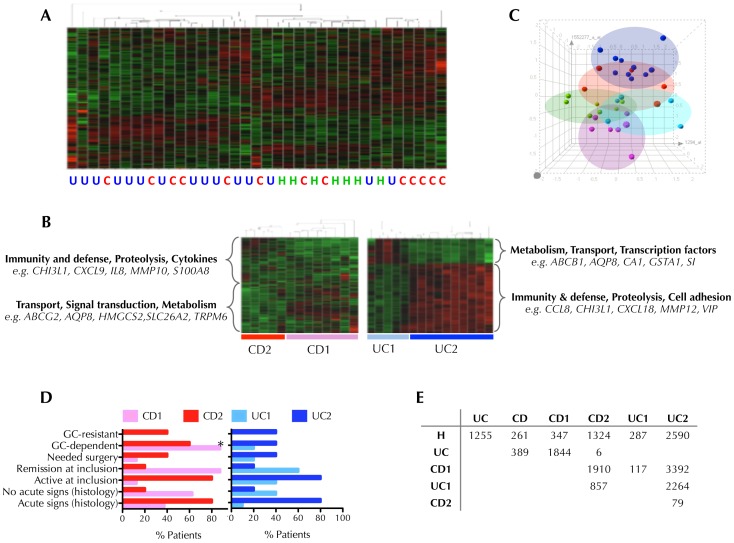
Discovery of Crohn’s disease (CD) and ulcerative colitis (UC) inflammatory subtypes by gene expression profiling. (A) Unsupervised hierarchical clustering of UC (blue), CD (red) and healthy controls (green) cases using the 54,675 probes contained in the chip. Up-regulated genes are shown in red and down-regulated genes in green. (B) Supervised hierarchical clustering of CD cases or UC cases using differentially expressed genes between CD and healthy (261 probes) and UC and healthy (1255 probes) respectively. This process defined two subgroups for both CD and UC cases. Functional analyses were performed using PANTHER Classification System. Examples of genes of each category are shown. (C) Clustering of samples using principal component analysis: CD1 (pink), CD2 (red), UC1 (light blue), UC2 (dark blue), healthy controls (green). (D) Association between clinical characteristics and CD and UC subtypes. Data represents the proportion of patients of each disease subtype included in different clinical variables, *p<0.05, Fisher exact test, CD1 *vs.* CD2 or UC1 *vs.* UC2. Complete clinical data is provided in Table S1. (E) Number of differentially expressed genes (*p*<0.05, t-test) in different comparisons among groups and with a fold change >1 or <−1.

An unsupervised hierarchical clustering using all genes represented in the chip was run in order to cluster the 35 subjects on the basis of their similarity and assess the degree of heterogeneity among samples. Although healthy samples seem to cluster together, the variation in gene expression patters in CD and UC was more complex, underlining the high degree of heterogeneity within IBD samples (Figure 1A). It is usually presumed that relatively few genes may distinguish between classes, and hence the differences between groups can be masked when using a whole genome clustering approach, as the vast majority of them would remain unchanged. Therefore, in order to study the heterogeneity within CD and UC patients, we performed a hierarchical clustering using the differentially expressed genes between CD and UC compared to healthy controls (261 and 1255 probes respectively). Surprisingly, in both cases samples were stratified in two distinct patient subgroups (Figure 1B) that were defined CD1 and UC1 as “Low Inflammation” subtypes and CD2 and UC2 as “High Inflammation” subtypes on the basis of the following findings. Functional annotation of the genes reveals up-regulation on metabolism or transport genes in the subgroups so-called CD1 and UC1, while up-regulation of predominantly inflammatory genes in the groups designated as CD2 and UC2. Principal component analysis (Figure 1C) also discriminated between the subgroups identified, and the association of these subgroups with clinical features further relates CD2 and UC2 with higher degree of disease activity (Figure 1D). Detailed clinical data is reported in Table S1. The number of differentially expressed genes between groups (Figure 1E) suggests an association between “more disease” profile with CD2 and UC2 as the number of altered genes for CD1 and UC1 respect to healthy controls was 347 and 287 probes respectively, but 1,324 for CD2 and 2,590 for UC2. The low number of differentially expressed genes between CD and UC (389) and between CD2 and UC2 (only 79) was truly striking, emphasizing the close pathogenic nature of CD and UC. The top 10 up- and down-regulated genes between CD1 *vs*. CD2 and UC1 *vs*. UC2 are reported in Table S3.

### Class-predictor analysis identifies sets of genes that discriminate between high and low inflammation subgroups of CD and UC

**Figure 2:**
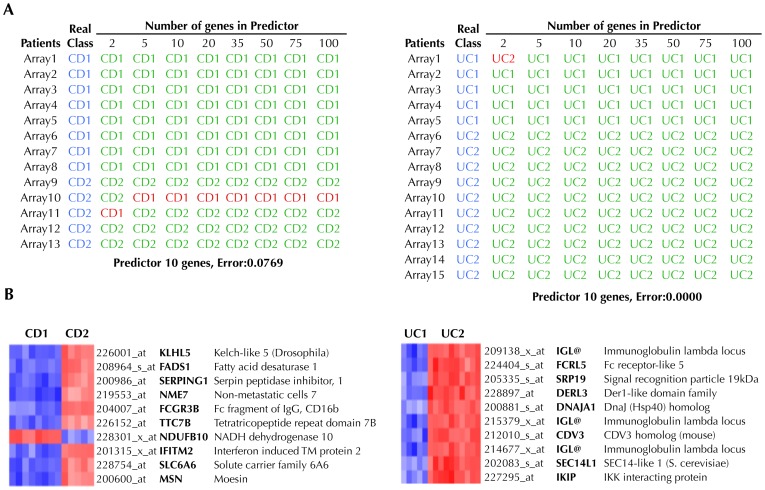
Identification of predictive classifiers of “High” and “Low” inflammation subtypes of Crohn’s disease (CD) and ulcerative colitis (UC). (A) A class predictor was built for both CD and UC using the tool Prophet and the 54,675 probes contained in the chip, using the leave-one-out cross-validation strategy. For each predictor, patients that were correctly classified are shown green, while those patients that the predictor failed to classify correctly are shown in red. (B) Based on the accuracy of each classifier, a 10-gene predictor was selected in both cases, which were able to accurately classify 92% of CD patients and 100% of UC patients. Up-regulated genes are shown in red and down-regulated in blue.

**Figure 3:**
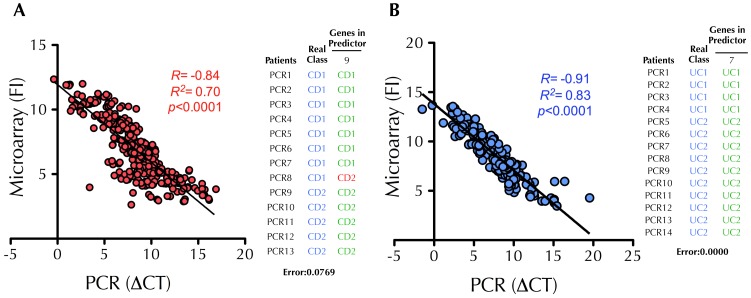
Confirmation of the predictive classifiers of “High” and “low” inflammation subtypes of Crohn’s disease (CD) and ulcerative colitis (UC) by quantitative real time-PCR. A total of 43 genes (including the predictors) were selected for real time-PCR validation of the microarray data in CD (A) and UC (B). Scatter plots show the correlation between microarray (fluorescence intensity, FI) and PCR (DCt value respect to the endogenous GAPDH, DCt) data, validating microarray results. The tables show the predictions obtained with the selected set of genes obtained from the microarray analysis using the PCR data. For each predictor, patients that were correctly classified are shown in green while those patients where the predictors failed to classify are shown in red. The classification accuracy obtained with PCR data was the same as the one obtained with the microarray data.

In order to discover new biomarkers related to the degree of intestinal inflammation we used the software package Prophet to identify the subset of genes that best discriminated CD1 from CD2 and UC1 from UC2, i.e. a predictor classifier of high and low inflammation activity. We searched for the smallest list of genes to accurately classify patients into their respective subgroups. Figure 2A shows the results from the cross-validation analysis. In the case of CD, there was a set of 5 genes that predicted if a sample belonged to group CD1 or CD2 with 92% accuracy. Interestingly, most of the predictors for UC had an accuracy of 100%. The expression of the genes included in the selected predictors (Figure 2B), was investigated in the public microarray repository GEO database. As shown in Table S4 the gene expression changes follow the same trend reported in other microarray experiments. Using this approach, previous reports confirmed the utility of class-predictor identification by running more microarray analysis in new patients [7]. However, the translation of these findings into clinical practice would require a more accessible technique than microarray profiling, such as quantitative real time-PCR. Therefore, a total of 42 genes (Table S2) were selected to analyze the general agreement between our microarray and PCR data. The correlation between both techniques was found to be optimal (Figure 3) with a correlation of 84% and 91% for CD and UC respectively. Then, to validate our predictors by using PCR data instead of microarray data, we performed the predictor analysis using Prophet and available primers (see Table S2). The results showed exactly the same degree of predictor accuracy (CD1 *vs.* CD2, 92.3%; UC1 *vs.* UC2, 100%) as obtained with microarray data. These data suggest that real time-PCR analyses performed on microarray-based predictors can replicate the results with similar accuracy and hence can be a simple, economical and reliable approach for the analysis of class predictors.

### Identification of new predictor genes associated with clinical parameters and IBD phenotypes

**Figure 4:**
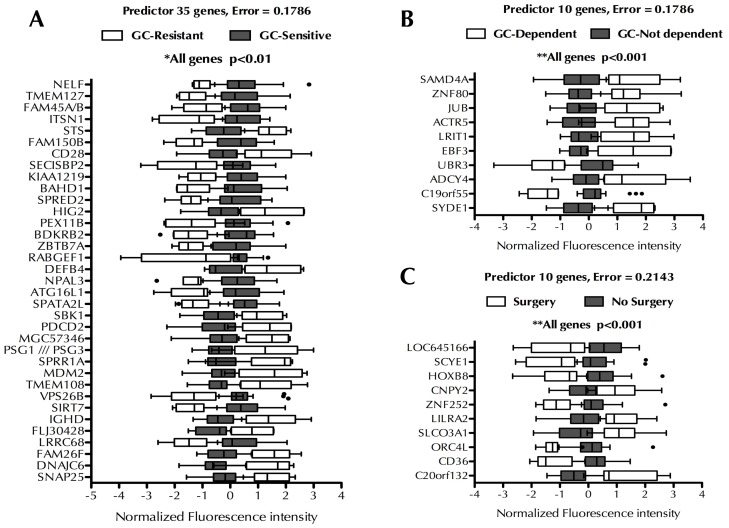
Systematic search for new predictors associated with clinical variables. Predictors were built using the tool Prophet and the 54,675 probes contained in the microarray, using the leave-one-out cross-validation strategy, to identify classifiers genes for glucocorticoid sensitivity (A), glucocorticoid dependency (B) or need for surgery (C). Data are represented as box and whiskers plots, where the error bars designate the smallest and largest observations and dots designate the outliers. Data was analyzed by two-way ANOVA followed by Bonferroni post test, *p* values higher than 0.05 were considered not significant (ns), *p<0.01, **p<0.001.

Once our approach had been validated, we focused on identifying new biomarkers related to relevant clinical parameters of IBD. Patients were classified according to glucocorticoid (GC) sensitivity, GC-dependency, IBD phenotype, if they had suffered IBD-related intestinal surgery or if they show high or low inflammation phenotype according to their gene expression profile. The statistical association among these subgroups of patients is shown in Figure S1. No significant association was observed among the different groups except for GC-sensitivity that was significantly associated with need of surgery and degree of inflammation. This result is not surprising because GC-resistance is usually associated with worse outcome and increased risk for surgery. Figure 4 summarizes the best sets of genes (i.e. sets with the fewest number of genes and the lowest prediction error) found to correctly classify patients according to GC-sensitivity (Figure 4A, 35 genes, 82% accuracy), GC-dependence (Figure 4B, 10 genes, 82% accuracy) and need of intestinal surgeries (Figure 4C, 10 genes, 79% accuracy). Two-way ANOVA with Bonferroni multiple testing correction was performed to assess if the difference in gene expression between the groups was significant. All genes showed a *p* value lower than 0.01.

**Figure 5:**
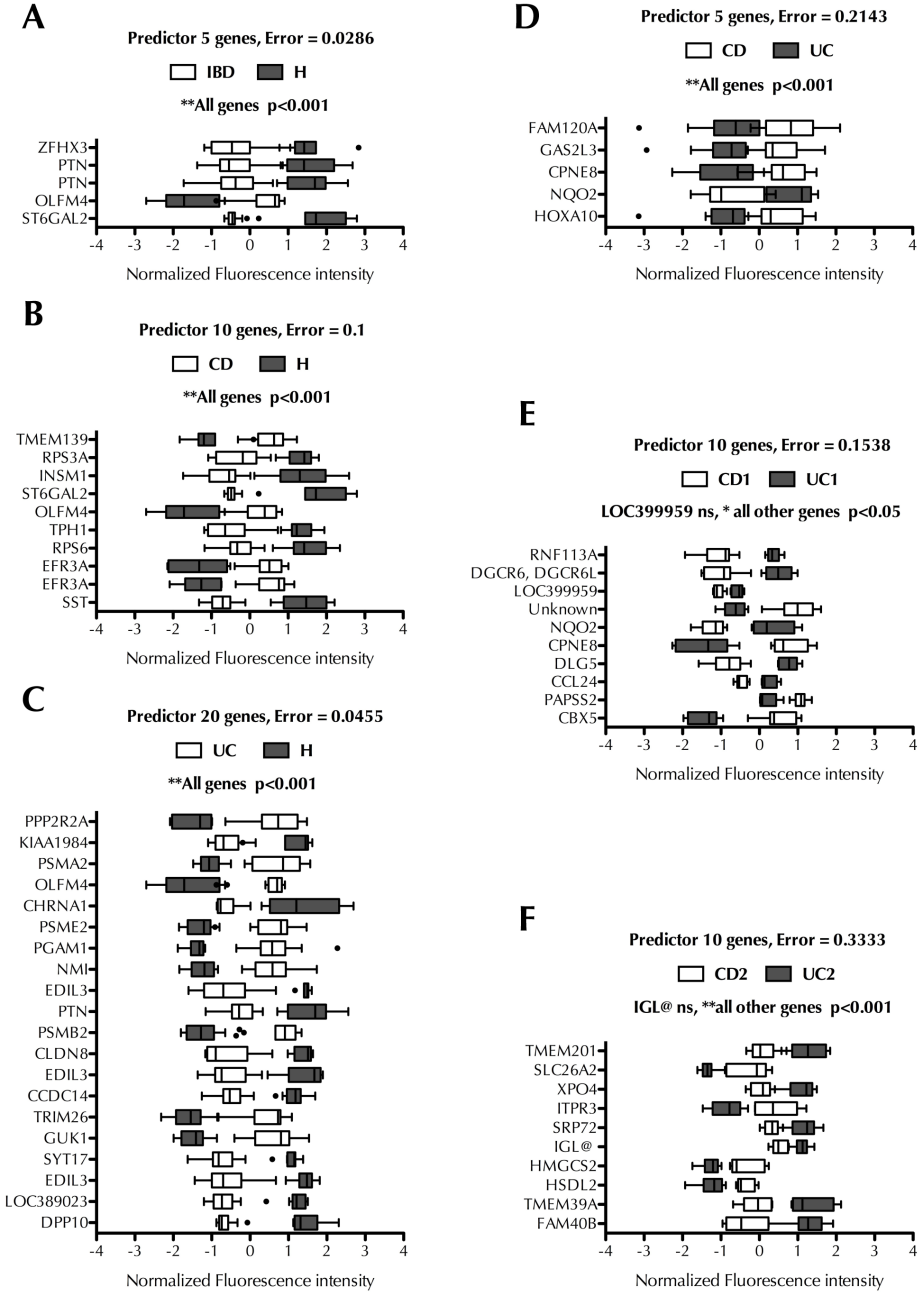
Systematic search for new predictors associated with IBD. Predictors were built using the tool Prophet and the 54,675 probes contained in the chip, using the leave-one-out cross-validation strategy, to identify classifiers genes for inflammatory bowel disease, IBD (A), Crohn’s disease, CD (B), ulcerative colitis, UC (C), CD *vs.* UC (D), low inflammation subtypes of CD and UC (E), and high inflammation subtypes of CD *vs.* UC (F). Data are represented as box and whiskers plots, where the error bars designate the smallest and largest observations and dots designate the outliers. Data was analyzed by two-way ANOVA followed by Bonferroni post test, *p* values higher than 0.05 were considered not significant (ns), *p<0.01, **p<0.001.

In the next step, we used this new approach to build predictor classifiers that would potentially aid in the accurate diagnosis of IBD. We built 5 different predictor classifiers performing the following comparisons: IBD (i.e. CD and UC) *vs*. H (healthy controls), CD *vs.* H, UC *vs.* H, CD *vs.* UC, CD1 *vs.* UC1 and CD2 *vs.* UC2. Figure 5 summarizes the best sets of genes found to correctly classify patients between these groups. The predictor classifiers of IBD forms *vs.* healthy samples showed the highest accuracy (>90%) but it dropped to 79% (CD *vs.* UC), 85% (CD1 *vs.* UC1) and 68% (CD2 *vs.* UC2). This result underlines the high similarity of both conditions at the gene expression level. The best predictor of CD *vs*. UC consisted of 5 genes with a prediction error of 21% (Figure 5D). A set of 10 genes was found to be the best predictor between the “High Inflammation” subgroups (CD2 and UC2) although the prediction error increased to 33% (Figure 5F). The lowest prediction error (15%) was obtained with “Low Inflammation” samples, i.e. CD1 *vs.* UC1 (Figure 5E). Interestingly, more than one probe for the same gene was identified in several predictor sets, a fact that may increase the significance of that particular gene as a potential biomarker. This was the case for the genes that encode for pleiotrophin (PTN), copine VIII (CPNE8), NAD(P)H dehydrogenase quinone 2 (NQO2) and EFR3 homolog A (EFR3A). OLFM4 and PTN were up-regulated in the predictor set that discriminated healthy controls from UC and CD patients (Figure 5A-C) (Figure 5D-F), suggesting a common biological role for OLFM4 and PTN in both forms of IBD. NQO2 and CPNE8 were identified as classifiers that discriminate CD *vs.* UC and CD1 *vs.* UC1 (Figure 5D and E), suggesting a potential utility as biomarkers for IBD diagnosis.

## Discussion

The ability to predict an individual’s clinical course is probably one of the major causes for concern for IBD patients, as it implies the appropriate choice of therapeutic tools. The response to therapy cannot be predicted at diagnosis and therapeutic approaches are individualized according to the symptomatic response and tolerance to medical intervention. Although existing serologic and fecal biomarkers can help diagnose and guide therapeutic choices [5] sensitivity, specificity and overall usefulness are not well established and their application in patient stratification is still debated. From the genetic perspective, a number of genetic markers have been associated with several clinical features, but to date, only the thiopurine S-methyltransferase genotyping test to assess toxicity of thiopurine has been translated into IBD clinical practice [12].

Whereas gene expression profiling has been successfully used to predict metastasis and response to chemotherapy in oncology, they have generally not detected signatures with equivalent prognostic value in IBD and low, if any, inter-translational coincidence between their transcriptional signatures can be found. Heterogeneous results may be due to confounding factors such as disease heterogeneity, racial lineage, variability in sample location, extraction procedure, cellular composition and differences in microarray platform and normalization algorithm. Despite these difficulties, efforts to reduce sample variability and the application of new tools and algorithms, that go far beyond the classical selection of a number of up- or down-regulated genes, to provide a small set of classifier genes may reliably translate mathematical and statistical analyses into clinical practice [13]. Indeed, Lee *et al* recently identified a classifier set of genes that predict prognosis in IBD patients [14]. CD and UC patients were stratified into two subgroups on the basis of CD8+ T cell gene expression profiling which correlated with the course of the disease.

Using the same strategy of class prediction, we set out to explore whether whole-gene expression profiling may provide clinically relevant biomarkers for IBD. This hypothesis aims at the selection of the smallest set of genes that accurately classified IBD subgroups using new *in silico* analyses. As in Lee *et al* [14], a hierarchical clustering of our IBD patients identified 2 distinct populations within CD and UC patients on the basis of their gene expression profile, which correlated with the degree of inflammation according to our functional analysis, clinical data and differentially expressed genes associated to each subgroup. It is interesting that our data confirm the existence of this gene expression signature associated with a more aggressive disease behavior. Our strategy was validated with the identification of the smallest set of genes that best discriminated between high (CD2, UC2) or low (CD1, UC1) inflammation phenotypes. A predictor set of 5 genes was identified that classified CD patients into high or low inflammation phenotypes with 92% of samples correctly classified. The positive and negative predictive values were 100 and 88% respectively; sensitivity was 80% and specificity 100%. In the case of UC, a 10-gene set was also identified as predictive for UC patients, with 100% sensitivity and specificity. Notably, none of these genes was found among the top up- or down-regulated genes with fold change (>1 or <−1) and *p*-value (<0.05). This observation indicates that the classical approach of selecting a list of differentially expressed genes for classification studies in IBD may not be appropriate and possibly explain the lack of success in defining and validating biomarkers. We also observed that, rather than a big difference (i.e. in fold change), the consistency among samples may be a more relevant parameter to identify a good biomarker, as most of the predictors discovered in this work showed a *p* value <0.001 despite a modest fold change. Equally important for a good biomarker is that it can be easily measured. We quantified the expression of 42 selected genes (including those in the predictor sets) in the 35 subjects included in this study (i.e. more than 1,400 measurements). The correlation between real time-PCR and microarray data was highly concordant (84% and 92% for CD and UC, respectively). This observation validated the accuracy of the gene expression data in our experimental procedure.

The high/low inflammation predictors can be informative and provide clues to IBD pathogenesis. Our aim, however, was to identify clinically relevant predictor genes to classify patients according to GC-sensitivity, GC-dependence, need for surgery and diagnosis. We successfully identified gene panels that discriminate between these conditions. In view of the potential heterogeneity and complexity of the mechanisms contributing to GC response and its potential impact on the natural history of IBD, it is noteworthy that pathway analysis identified autophagy and endocytosis processes in the 35 genes set of GC-sensitivity predictor (sensitivity and specificity of 33% and 95% respectively). ATG16L1 along with other 5 genes are involved in endocytosis processes (DNAJC6, VPS26B, RABGEF1, ITSN1 and TMEM127). ATG16L1, a reported susceptibility gene for IBD, controls autophagy, a crucial process in the resistance against infection and removal of intracellular microbes. Interestingly, Bonapace *et al* found that activation of autophagy was able to resensitize GC-resistant leukemia cells [15]. Accordingly, ATG16L1 was down-regulated in GC-resistant patients, which might lead us to speculate that induction of autophagy might help to overcome GC-resistance in IBD patients, as it happens in leukemia. Other genes encoding for steroid sulfatase (STS) and MDM2 oncogene E3 ubiquitin protein ligase, both involved in the proteosomal degradation of the GC receptor [16], may also play a role in the mechanisms leading to GC-resistance. CD36 and LILRA2 (leukocyte immunoglobulin-like receptor, subfamily A with TM domain, member 2) were included in the predictor set for need of surgery (sensitivity and specificity of 50% and 85% respectively). Deficiency of CD36 scavenger receptor, mainly expressed in macrophages, has been associated with a more aggressive colitis in mice [17]. LILRA2 up-regulation is also worth mentioning as this protein induces cytokine production by monocytes while reducing their phagocytic ability [18] and correlates with disease severity in rheumatoid arthritis [19]. The best predictor from our study was able to distinguish IBD from healthy subjects with 97% accuracy with only 5 probes (sensitivity and specificity of 100% and 86% respectively), 2 of them representing the gene PTN (pleiotrophin) which has already been described to be down-regulated in CD patients [20]. Pleiotrophin controls several processes such as angiogenesis or cell proliferation but it also exerts strong antibacterial activity [21], suggesting another innate immune mechanism deregulated in IBD. The same accounts for the glycoprotein olfactomedin 4 (OLFM4), up-regulated in both forms of IBD according to our data, that is secreted into mucus [22] and acts by inhibiting immune responses against *Helicobacter pylori*, hence contributing to the persistence of infection by a mechanism involving NOD1 and NOD2 [23].

Another ramification of our study was to identify predictor sets that would discriminate between CD and UC. The accuracy dropped when we built predictor sets to discriminate between CD and UC. While this result may appear unsatisfactory, the predictive capacity of our predictor sets (Figure 5 D-E-F, from 68% to 84%) is at the level of those proposed by Hakonarson *et al* to predict GC response in asthma patients [24]. Indeed, prognosis in patients with CD and UC was proposed on the basis of predictor signatures with 59% (CD) and 77% (UC) sensitivities [14]. The fact that the comparison of low inflammation groups CD1 *vs.* UC1 yielded better predictors than high inflammation groups CD2 *vs.* UC2 suggests that the inflammatory process at active stages is a natural confounding factor that limits the ability of the predictor set. In this case, gene expression analysis of predictor genes in patients in remission may therefore be preferential for a better diagnosis of IBD.

In summary, we show here that gene expression profiling can be successfully used to stratify patients and identify transcriptional signatures associated with clinical parameters. We identified several predictor gene panels that contained genes involved in immune mechanisms (PTN, OLFM4, LILRA2, CD36), autophagy or GC response (STS, MDM2). This represents, to our knowledge, the first biomarker discovery based on specifically designed analytical algorithms with potential value to predict GC response and need of surgery as well as with diagnostic value for IBD patients. This could therefore enable patients to be classified and receive personalized therapy according to the expression level of predictor genes. Our study is the first stone in the validation of the potential use of our class predictors in larger cohorts of IBD patients.

## Supporting Information

Figure S1 **Association between the different patient subgroups used for the predictor analyses.** Data were analyzed by Fisher’s exact test and *p* values lower that 0.05 were considered significant.
(TIFF)

Table S1 Association between clinical parameters and inflammatory profile.
(DOC)

Table S2 Top 10 up-regulated and down-regulated genes between ulcerative colitis subtypes (UC1 *vs.* UC2) and Crohn's disease subtypes (CD1 *vs.* CD2).
(DOC)

Table S3 Genes validated by real time-PCR and primers used.
(DOC)

Table S4 Gene expression changes identified in GEO Profiles database.
(DOCX)
