# Supplementary figures and images for: Identification of Novel Predictor Classifiers for Inflammatory Bowel Disease by Gene Expression Profiling
Source: PLoS One. 2013 Oct 14;8(10):e76235. doi: 10.1371/journal.pone.0076235 (PMC3796518; doi:10.1371/journal.pone.0076235)

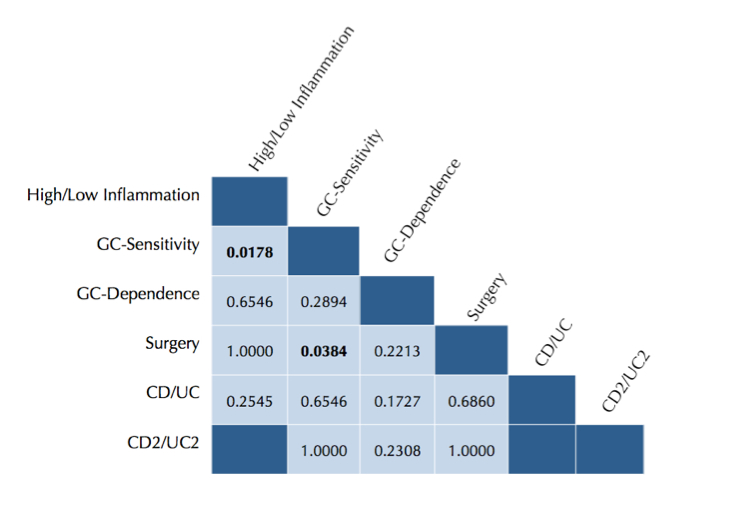

Supplement: Figure S1 — Association between the different patient subgroups used for the predictor analyses. Data were analyzed by Fisher’s exact test and p values lower that 0.05 were considered significant. (TIFF) [file pone.0076235.s001.tiff]
