# Supplementary material for: Identification of Novel Predictor Classifiers for Inflammatory Bowel Disease by Gene Expression Profiling
Source: PLoS One. 2013 Oct 14;8(10):e76235. doi: 10.1371/journal.pone.0076235 (PMC3796518; doi:10.1371/journal.pone.0076235)
Supplement: Table S1 — Association between clinical parameters and inflammatory profile. (DOC) [file pone.0076235.s002.doc]

|  | | **Ulcerative colitis** | | | **Crohn’s Disease** | | |
| --- | --- | --- | --- | --- | --- | --- | --- |
| **Characteristic** | | **High Inflammation Profile UC2 (n=10)** | **Low Inflammation Profile UC1**  **(n=5)** | **P value** | **High Inflammation Profile CD2 (n=5)** | **Low Inflammation Profile CD1**  **(n=8)** | **P value** |
| **Sex** | Male | 4 (40%) | 3 (60%) | 0.6084 | 4 (80%) | 5 (62.5%) | 1.0000 |
|  | Female | 6 (60%) | 2 (40%) |  | 1 (20%) | 3 (37.5%) |  |
| **Age** | ≤30 | 2 (20%) | 1 (20%) | 0.7502 | 1 (20%) | 0 (0%) | 0.4594 |
|  | 31-60 | 7 (70%) | 3 (60%) |  | 2 (40%) | 8 (100%) |  |
|  | >60 | 1 (10%) | 1 (20%) |  | 2 (40%) | 0 (0%) |  |
| **Age at diagnosis** | ≤30 | 6 (60%) | 3 (60%) | 0.5548 | 2 (40%) | 5 (62.5%) | 0.2399 |
|  | 31-60 | 4 (40%) | 1 (20%) |  | 2 (40%) | 3 (37.5%) |  |
|  | >60 | 0 (0%) | 1 (20%) |  | 1 (10%) | 0 (0%) |  |
| **Activity at inclusion** | Active | 8 (80%) | 2 (40%) | 0.2507 | 4 (80%) | 1 (12.5%) | 0.0319* |
|  | Remission | 2 (20%) | 3 (60%) |  | 1 (20%) | 7 (87.5%) |  |
| **Extension** | Extensa-Pancolitis | 5 (50%) | 4 (80%) | 0.4318 | -- | -- | 0.4330 |
|  | Left side colon | 1 (10%) | 1 (20%) |  | -- | -- |  |
|  | Proctitis | 3 (30%) | 0 (0%) |  | -- | -- |  |
|  | Angulo esplenico | 1 (10%) | 0 (0%) |  | -- | -- |  |
|  | Colon | -- | -- |  | 3 (60%) | 3 (37.5%) |  |
|  | Colon+Ileum | -- | -- |  | 1 (20%) | 3 (37.5%) |  |
|  | Colon+EGD | -- | -- |  | 1 (20%) | 0 (0%) |  |
|  | Ileum | -- | -- |  | 0 (0%) | 1 (12.5%) |  |
|  | Not confirmed | -- | -- |  | 0 (0%) | 1 (12.5%) |  |
| **Co-morbidities** | Yes | 3 (30%) | 1 (20%) | 1.0000 | 3 (60%) | 5 (62.5%) | 1.0000 |
|  | No | 7 (70%) | 4 (80%) |  | 2 (40%) | 3 (37.5%) |  |
| **Needed surgery** | Yes | 4 (40%) | 1 (20%) | 0.6004 | 2 (40%) | 1 (12.5%) | 0.5105 |
|  | No | 6 (60%) | 4 (80%) |  | 3 (60%) | 7 (87.5%) |  |
| **Histology** | Acute | 8 (80%) | 1 (10%) | 0.0889 | 4 (80%) | 3 (37.5%) | 0.2657 |
|  | Chronic | 2 (20%) | 4 (40%) |  | 1 (20%) | 5 (62.5%) |  |
| **Smoker** | Yes | 1 (10%) | 0 (0%) | 1.0000 | 1 (20%) | 3 (37.5%) | 1.0000 |
|  | No | 9 (90%) | 5 (100%) |  | 4 (80%) | 5 (62.5%) |  |
| **Dysplasia** | Yes | 0 (0%) | 0 (0%) | 1.0000 | 1 (20%) | 0 (0%) | 0.3846 |
|  | No | 10 (100%) | 5 (100%) |  | 4 (80%) | 8 (100%) |  |
| **GC-Resistant** | Yes | 4 (40%) | 0 (0%) | 0.2308 | 2 (40%) | 0 (0%) | 0.1282 |
|  | No | 6 (60%) | 5 (100%) |  | 3 (60%) | 8 (100%) |  |
| **GC-Dependent** | Yes | 4 (40%) | 1 (20%) | 0.6004 | 0 (0%) | 1 (12.5%) | 1.0000 |
|  | No | 6 (60%) | 4 (80%) |  | 5 (100%) | 7 (87.5%) |  |
| **Anti-TNFα** | Yes | 5 (50%) | 1 (20%) | 0.5804 | 2 (40%) | 4 (50%) | 1.0000 |
|  | No | 5 (50%) | 4 (80%) |  | 3 (60%) | 4 (50%) |  |
| **Metotrexate** | Yes | 1 (10%) | 0 (0%) | 1.0000 | 1 (20%) | 3 (37.5%) | 1.0000 |
|  | No | 9 (90%) | 5 (100%) |  | 4 (80%) | 5 (62.5%) |  |
| **Years with disease** | ≤10 | 7 (70%) | 3 (60%) | 1.0000 | 5 (100%) | 3 (37.5%) | 0.0754 |
|  | >10 | 3 (30%) | 2 (40%) |  | 0 (0%) | 5 (62.5%) |  |
| **Glucocorticoids** | YES | 8 (80%) | 1 (20%) | 0.0889 | 2 (40%) | 1 (12.5%) | 0.5105 |
|  | NO | 2 (20%) | 4 (80%) |  | 3 (60%) | 7 (87.5%) |  |

Table S1
