# Supplementary material for: Identification of Novel Predictor Classifiers for Inflammatory Bowel Disease by Gene Expression Profiling
Source: PLoS One. 2013 Oct 14;8(10):e76235. doi: 10.1371/journal.pone.0076235 (PMC3796518; doi:10.1371/journal.pone.0076235)
Supplement: Table S2 — Top 10 up-regulated and down-regulated genes between ulcerative colitis subtypes (UC1 vs. UC2) and Crohn's disease subtypes (CD1 vs. CD2). (DOC) [file pone.0076235.s003.doc]

| **Gene Symbol** | **Primers** | **Name** |
| --- | --- | --- |
| **ABCG2** | QT00073206 | ATP-binding cassette, sub-family G, member 2 |
| **ANPEP** | QT00000805 | Alanyl (membrane) aminopeptidase |
| **AQP8** | QT00039123 | Aquaporin 8 |
| **C3** | QT01680413 | Complement component 3 |
| **CA1** | QT00006440 | Carbonic anhydrase I |
| **CDV3 *** | QT00095368 | Carnitine deficiency-associated gene expressed in ventricle 3 |
| **CHI3L1** | QT00066703 | Chitinase 3-like 1 |
| **CXCL11** | QT02394644 | Chemokine (C-X-C motif) ligand 11 |
| **CXCL13** | QT00017129 | Chemokine (C-X-C motif) ligand 13 |
| **CXCR4** | QT02311841 | Chemokine (C-X-C motif) receptor 4 |
| **DERL3 *** | QT00494473 | Der1-like domain family, member 3 |
| **DNAJA1 *** | QT01674701 | DnaJ (Hsp40) homolog, subfamily A, member 1 |
| **DUOX2** | QT00012236 | Dual oxidase 2 |
| **DUOXA2** | QT00074515 | Dual oxidase maturation factor 2 |
| **FADS1 *** | QT02322621 | Fatty acid desaturase 1 |
| **FCRL5 *** | QT00062517 | Fc receptor-like 5 |
| **GAPDH** | QT01192646 | Glyceraldehyde-3-phosphate dehydrogenase |
| **GREM1** | QT01018633 | Gremlin 1 |
| **HMGCS2** | QT00011389 | 3-hydroxy-3-methylglutaryl-CoA synthase 2 |
| **IFITM2 *** | QT01883413 | Interferon induced transmembrane protein 2 |
| **IKIP *** | QT01672902 | IKBKB interacting protein |
| **IL8** | QT00000322 | Interleukin 8 |
| **KLHL5 *** | QT00046179 | Kelch-like 5 |
| **KYNU** | QT00084252 | Kynureninase (L-kynurenine hydrolase) |
| **MMP10** | QT00001470 | Matrix metallopeptidase 3 |
| **MMP3** | QT00060025 | Matrix metallopeptidase 3 |
| **MSN *** | QT00015169 | Moesin |
| **NDUFB10 *** | QT00237426 | NADH dehydrogenase (ubiquinone) 1 beta subcomplex, 10 |
| **NME7 *** | QT00039802 | Non-metastatic cells 7 |
| **REG1B** | QT01667337 | Regenerating islet-derived 1 beta |
| **REG3A** | QT01005543 | Regenerating islet-derived 3 alpha |
| **RSPO3** | QT00025879 | R-spondin 3 homolog |
| **S100A8** | QT00226121 | S100 calcium binding protein A8 |
| **SEC14L1 *** | QT00076377 | SEC14-like 1 |
| **SERPING1 *** | QT00053963 | Serpin peptidase inhibitor, clade G (C1 inhibitor), member 1 |
| **SLC16A1** | QT00012838 | Solute carrier family 16, member 1 |
| **SLC26A2** | QT01851864 | Solute carrier family 26, member 2 |
| **SLC6A14** | QT00087542 | Solute carrier family 6, member 14 |
| **SLC6A6 *** | QT00095655 | Solute carrier family 6, member 6 |
| **SRP19 *** | QT00076790 | Signal recognition particle 19kDa |
| **TTC7B *** | QT00088305 | Tetratricopeptide repeat domain 7B |
| **UGT1A6** | QT01668422 | UDP glucuronosyltransferase 1 family, polypeptide A6 |
| **VNN1** | QT00004165 | Vanin 1 |

Table 2

* Predictor genes validated by real time-PCR.
