# Supplementary material for: Identification of Novel Predictor Classifiers for Inflammatory Bowel Disease by Gene Expression Profiling
Source: PLoS One. 2013 Oct 14;8(10):e76235. doi: 10.1371/journal.pone.0076235 (PMC3796518; doi:10.1371/journal.pone.0076235)
Supplement: Table S3 — Genes validated by real time-PCR and primers used. (DOC) [file pone.0076235.s004.doc]

| **UC1 *vs.* UC2 (2264 probes)** | | | | |
| --- | --- | --- | --- | --- |
| **Probe Set ID** | **Gene Symbol** | **Gene Title** | **Fold Change** | ***p* value** |
| 208383_s_at | **PCK1** | Phosphoenolpyruvate carboxykinase 1 | **5.04** | **0.000** |
| 206784_at | **AQP8** | Aquaporin 8 | **4.78** | **0.002** |
| 209735_at | **ABCG2** | ATP-binding cassette, sub-family G (WHITE), member 2 | **4.69** | **0.000** |
| 210445_at | **FABP6** | Fatty acid binding protein 6, ileal | **4.30** | **0.006** |
| 207003_at | **GUCA2A** | Guanylate cyclase activator 2A (guanylin) | **4.17** | **0.000** |
| 222943_at | **GBA3** | Glucosidase, beta, acid 3 (cytosolic) | **4.13** | **0.000** |
| 202888_s_at | **ANPEP** | Alanyl (membrane) aminopeptidase | **4.08** | **0.000** |
| 229230_at | **OSTalpha** | Organic solute transporter alpha | **4.03** | **0.001** |
| 204607_at | **HMGCS2** | 3-hydroxy-3-methylglutaryl-Coenzyme A synthase 2 | **3.92** | **0.001** |
| 207245_at | **UGT2B17** | UDP glucuronosyltransferase 2 family, polypeptide B17 | **3.88** | **0.016** |
|  |  |  |  |  |
| 203915_at | **CXCL9** | Chemokine (C-X-C motif) ligand 9 | **-3.90** | **0.000** |
| 219795_at | **SLC6A14** | Solute carrier family 6 (amino acid transporter), member 14 | **-3.98** | **0.001** |
| 211122_s_at | **CXCL11** | Chemokine (C-X-C motif) ligand 11 | **-4.12** | **0.000** |
| 202859_x_at | **IL8** | Interleukin 8 | **-4.15** | **0.000** |
| 218469_at | **GREM1** | Gremlin 1, cysteine knot superfamily | **-4.30** | **0.000** |
| 205680_at | **MMP10** | Matrix metallopeptidase 10 (stromelysin 2) | **-4.52** | **0.000** |
| 218468_s_at | **GREM1** | Gremlin 1, cysteine knot superfamily | **-4.70** | **0.000** |
| 205828_at | **MMP3** | Matrix metallopeptidase 3 (stromelysin 1, progelatinase) | **-5.48** | **0.000** |
| 209396_s_at | **CHI3L1** | Chitinase 3-like 1 (cartilage glycoprotein-39) | **-5.71** | **0.000** |
| 209395_at | **CHI3L1** | Chitinase 3-like 1 (cartilage glycoprotein-39) | **-5.85** | **0.000** |
|  |  |  |  |  |
| **CD1 *vs.* CD2 (1910 probes)** | | | | |
| **Probe Set ID** | **Gene Symbol** | **Gene Title** | **Fold Change** | ***p* value** |
| 229230_at | **OSTalpha** | Organic solute transporter alpha | **5.08** | **0.000** |
| 206784_at | **AQP8** | Aquaporin 8 | **4.64** | **0.000** |
| 214598_at | **CLDN8** | Claudin 8 | **4.25** | **0.000** |
| 204607_at | **HMGCS2** | 3-hydroxy-3-methylglutaryl-Coenzyme A synthase 2 | **3.73** | **0.000** |
| 209735_at | **ABCG2** | ATP-binding cassette, sub-family G (WHITE), member 2 | **3.55** | **0.002** |
| 208383_s_at | **PCK1** | Phosphoenolpyruvate carboxykinase 1 (soluble) | **3.49** | **0.000** |
| 240110_at | **HMGCS2** | 3-hydroxy-3-methylglutaryl-Coenzyme A synthase 2 | **3.40** | **0.000** |
| 207003_at | **GUCA2A** | Guanylate cyclase activator 2A (guanylin) | **3.19** | **0.001** |
| 207502_at | **GUCA2B** | Guanylate cyclase activator 2B (uroguanylin) | **3.04** | **0.001** |
| 220786_s_at | **SLC38A4** | Solute carrier family 38, member 4 | **3.03** | **0.000** |
|  |  |  |  |  |
| 205815_at | **REG3A** | Regenerating islet-derived 3 alpha | **-4.36** | **0.022** |
| 205828_at | **MMP3** | Matrix metallopeptidase 3 (stromelysin 1, progelatinase) | **-4.44** | **0.000** |
| 209752_at | **REG1A** | Regenerating islet-derived 1 alpha | **-4.44** | **0.004** |
| 218468_s_at | **GREM1** | Gremlin 1, cysteine knot superfamily | **-4.53** | **0.000** |
| 202917_s_at | **S100A8** | S100 calcium binding protein A8 | **-4.56** | **0.000** |
| 205886_at | **REG1B** | Regenerating islet-derived 1 beta | **-4.71** | **0.004** |
| 219727_at | **DUOX2** | Dual oxidase 2 | **-4.83** | **0.001** |
| 219795_at | **SLC6A14** | Solute carrier family 6 (amino acid transporter), member 14 | **-5.10** | **0.000** |
| 209396_s_at | **CHI3L1** | Chitinase 3-like 1 (cartilage glycoprotein-39) | **-5.85** | **0.000** |
| 209395_at | **CHI3L1** | Chitinase 3-like 1 (cartilage glycoprotein-39) | **-6.62** | **0.000** |

Table 3
