# Supplementary material for: Identification of Novel Predictor Classifiers for Inflammatory Bowel Disease by Gene Expression Profiling
Source: PLoS One. 2013 Oct 14;8(10):e76235. doi: 10.1371/journal.pone.0076235 (PMC3796518; doi:10.1371/journal.pone.0076235)
Supplement: Table S4 — Gene expression changes identified in GEO Profiles database. (DOCX) [file pone.0076235.s005.docx]

| **Gene symbol** | **Observation** | **GEO Profile accession number** | **Gene ID-REF** |
| --- | --- | --- | --- |
| CDV3 | Increased in UC (inflamed *vs.* non-inflamed) | GDS3119 | 213548_s_at |
| DERL3 | Increased in UC (inflamed *vs.* non-inflamed) | GDS3119 | 229721_at |
| FADS1 | Increased in CD | GDS1330 | 42N03 |
| FCGR3B | Increased in UC (inflamed *vs.* non-inflamed) | GDS3119 | 204007_at |
|  | Increased in UC vs. irritable bowel syndrome | GDS2014 | 204007_at |
| FCRL5 | Increased in UC (inflamed *vs.* non-inflamed) | GDS3119 | 224405_at |
| IFITM2 | Increased in UC | GDS3119 | 201315_at |
| IGL@ | Increased in UC | GDS3119 | 215214_at |
| IKIP | Increased in UC (inflamed *vs.* non-inflamed) | GDS3119 | 227295_at |
| KLH5 | Increased in CD and UC | GDS1330 | 67N22 |
|  | Increased in UC | GDS3119 | 226001_at |
| MSN | Increased in UC | GDS3119 | 200600_at |
| NDUFB10 | Decreased in UC (inflamed *vs.* non-inflamed) | GDS3119 | 228301_x_at |
|  | Decreased in CD | GDS560 | 223112_s_at |
| NME7 | Increased in CD and UC | GDS560 | 227556_at |
| SEC14L | Increased in UC (inflamed *vs.* non-inflamed) | GDS3119 | 202082_s_at |
| SLC6A6 | Increased in UC | GDS3119 | 228754_at |
| SRP19 | Increased in UC (inflamed *vs.* non-inflamed) | GDS3119 | 205335_s_at |
| TTC7B | Increased in UC | GDS3119 | 226152_at |

Table S4
